# Induction of Sublethal Oxidative Stress on Human Sperm before Cryopreservation: A Time-Dependent Response in Post-Thawed Sperm Parameters

**DOI:** 10.22074/cellj.2019.5639

**Published:** 2018-08-07

**Authors:** Maryam Hezavehei, Homa Mohseni Kouchesfahani, Abdolhossein Shahverdi, Mohsen Sharafi, Ghasem Hosseini Salekdeh, Poopak Eftekhari-Yazdi

**Affiliations:** 1 Department of Animal Biology, Faculty of Biological Sciences, Kharazmi University, Tehran, Iran; 2 Department of Embryology, Reproductive Biomedicine Research Centre, Royan Institute for Reproductive Biomedicine, ACECR, Tehran, Iran; 3 Department of Poultry Science, College of Agriculture, Tarbiat Modarres University, Tehran, Iran; 4 Department of Molecular Systems Biology, Cell Science Research Centre, Royan Institute for Stem Cell Biology and Technology, ACECR, Tehran, Iran

**Keywords:** Cryotolerance, Freezing, Nitric Oxide, Preconditioning, Sperm

## Abstract

**Objective:**

A recent innovative approach, based on induction of sublethal oxidative stress to enhance sperm cryosurvival, has been applied before sperm cryopreservation. The purpose of this study was to investigate the effects of different induction times of sublethal oxidative stress before cryopreservation on human post-thawed sperm quality.

**Materials and Methods:**

In this experimental study, we selected semen samples (n=20) from normozoospermic men according to 2010 World Health Organization (WHO) guidelines. After processing the samples by the density gradient method, we divided each sample into 5 experimental groups: fresh, control freezing, and 3 groups exposed to 0.01 μM sodium nitroprusside (SNP) [nitric oxide (NO) donor] for 30 (T30), 60 (T60), or 90 minutes (T90) at 37˚C and 5% CO_2_ before cryopreservation. Motion characteristics [computer-assisted sperm analyser], viability, apoptosis [annexin V/propidium iodide (PI) assay], DNA fragmentation [sperm chromatin structure assay (SCSA)], and caspase 3 activity (FLICA Caspase Detection Kit) were assessed after thawing. The results were analysed by using one-way ANOVA and Tukey’s test. The means were significantly different at P<0.05.

**Results:**

Cryopreservation significantly decreased sperm viability and motility parameters, and increased the percentage of apoptosis, caspase 3 activity, and DNA fragmentation (P<0.01) compared to the fresh group. The T60 group had a higher significant percentage of total motility (TM) and progressive motility compared with other cryopreserved groups (P<0.05). We observed a significantly lower percentage of apoptotic rate and caspase 3 activity in the T60 group compared to the other cryopreserved groups (P<0.05). DNA integrity was not significantly affected by this time of sublethal stress induction (P>0.05).

**Conclusion:**

Our results have demonstrated that the application of sublethal oxidative stress by using 0.01 μM NO for 60 minutes before the freezing process can be a beneficial approach to improve post-thawed human sperm quality.

## Introduction

Sperm cryopreservation is a beneficial approach for conservation of male fertility (1). However, in the cryopreservation process, physical and biochemical stresses can impact sperm quality which leads to a loss of its viability and fertilization potential (2). This phenomenon is largely due to high production of reactive oxygen species (ROS) and ice crystal formation (3), with eventual destruction of the plasma membrane and DNA integrity (4, 5). Therefore it is necessary to optimize a strategy to reduce these cryodamages (6). 

In recent years, a controllable sublethal oxidative stress has been applied for cryopreservation of semen (7), oocytes (8), and embryos (9). Researchers proposed that the application of sublethal stresses such as high hydrostatic pressure (HHP) (10, 11), osmotic pressure (12, 13), ethanol (14), and oxidative agents (15) before cryopreservation could lead to enhanced resistance of sperm against cryodamage. Huang et al. (10) reported that post-thaw motility of boar treated sperm with HHP increased compared to the control as result of alterations in the protein profile of the sperm. In a recent study, increased phosphorylation of heat shock proteins (HSP) of treated macaque sperm with osmotic stress led to improved cryosurvival (12). Animal studies showed the positive effects of mild stress on improved sperm function by reducing lipid peroxidation and increasing motility (14, 15) . However, the time of the stress induction before cryopreservation was a key factor on the effectiveness of the sublethal stress (13). Different induction times for sublethal stress showed various responses in adaptation and increased resistance of sperm against freezing-thawing (7). Accordingly, it has been reported that application of mild oxidative stress at 120 and 45 minutes of cooling period before freezing improved the quality of bull sperm after thawing (16). The present study aimed to investigate the effects of different induction times of sublethal oxidative stress before human sperm cryopreservation on post-thawed sperm function.

## Materials and Methods

All chemicals used in this study were purchased from Sigma-Aldrich (St. Louis, MO, USA) unless otherwise mentioned. The Research Ethics Committees of Royan Institute approved this study (IR. ACER. ROYAN. REC. 1396. 80). We received all the patients consents.

### Semen collection and study design

This was an interventional experimental study. A total of 20 normozoospermic men (age 31-38 years) provided semen samples by masturbation after 3 to 7 days of sexual abstinence. The exclusion criteria were: leukocytospermia (=1×10^6^ white blood cells/mL), varicocele or endocrine disorders, use of medication/antioxidants, and exposure to chemotherapy or radiation. Semen samples were accepted for this study according to 2010 World Health Organization (WHO) guidelines that included: sperm counts >34×10^6^ spermatozoa/mL, motility >40%, and leukocytes <1×10^6^/mL). Each sample was processed by a discontinuous Percoll gradient [45% and 90% All Grad (v/v); AGSS Life Global, Brussels, Belgium] and then diluted with human tubal fluid (HTF) medium supplemented with 5% human serum albumin (HSA). Subsequently, we divided each sample into 5 equal parts: fresh, control freezing (without treatment), and 3 groups exposed to 0.01 µM of the nitric oxide (NO) donor, sodium nitroprusside (SNP), at 30 (T30), 60 (T60), or 90 (T90) minutes before cryopreservation. We selected the concentration of 0.01 µM SNP on the basis of our preliminary study (data not published).

### Sperm cryopreservation 

We added freezing medium (FertiPro N.V., Beernem, Belguim) droplets to the sperm suspensions at a ratio of 0.7:1 v/v according to the manufacturer’s instructions for rapid freezing. After equilibration at room temperature, we transferred 1.0 ml of the mixtures into the cryotubes (Nunc, Roskilde, Denmark) for a final sperm concentration of approximately 5×10^6^. The cryotubes were exposed to a liquid nitrogen vapour-phase (10-15 cm above the level of the liquid nitrogen at-80°C) for 15 minutes, after which they were completely immersed in liquid nitrogen. After 7 days of storage in liquid nitrogen, the samples were thawed in a 37°C water bath for 5 minutes. The freezing medium was removed by the addition of 5 mL HTF medium that contained 5% HSA (Life Global, Guelph, ON, Canada). The samples were centrifuged; the pellets were re-suspended in the same medium, and incubated at 37°C in 5% CO_2_ for 15 minutes before evaluation.

### Assessment of sperm parameters after the freezing- thawing process

#### Motility and velocity parameters

Sperm motion parameters were determined using a computer-assisted sperm analyser (CASA, version 5.1; Microptic, Barcelona, Spain). We loaded 5 µL of the sperm suspension onto a pre-warmed chamber (20 µm, Leja 4, Leja Products Luzernestraat B.V., Holland). A minimum of 5 fields per sample were evaluated by the CASA program consisted of total motility (TM, %), progressive motility (PM, %), average path velocity (VAP, µm/sec), straight line velocity (VSL, µm/sec), curvilinear velocity (VCL, µm/sec), amplitude of straightness (STR, %), and linearity (LIN, %).

#### Phosphatidylserine translocation

Annexin V staining of membrane phosphatidylserine along with propidium iodide (PI) were used to determine the amount of apoptosis in the sperm cells for the different experimental groups. 

We added 10 µl of Annexin V-FITC to the washed sperms (1×10^6^ cells/mL) with calcium buffer, which was maintained for 15 minutes at room temperature. Then, we added 10 µl of PI to the sperm suspension and subseqently evaluated the percentages of live (annexin^-^/ PI), apoptotic (annexin^+^/PI), dead (annexin^+^/ PI^+^), and necrotic (annexin^-^/PI^+^) sperm with a FACSCalibur Flow cytometer (Becton Dickinson, San Jose, CA, USA). Green fluorescence emission for Annexin V (530/30 band pass) and red fluorescence emission for PI (610/20 band pass) were measured in the FL-1 and FL-3 channels, respectively (17). 

#### DNA fragmentation

**Fig.1:**
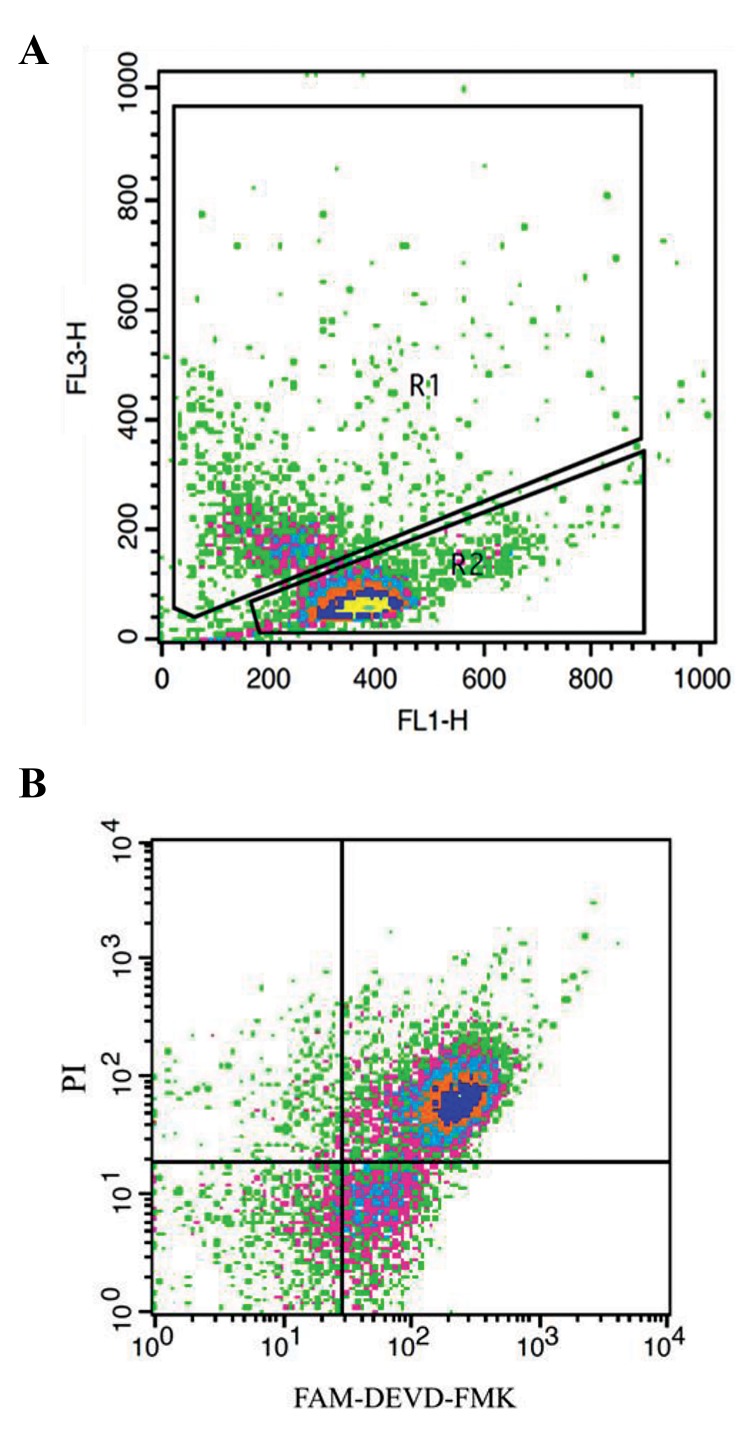
FloCytogram from analysis of 106 spermatozoa by sperm chromatin structure assay (SCSA) and caspase activity of spermatozoa with FAM- DEVD-FMK/propidium iodide (PI). A. Density plot of sperm cells by SCSA FL1: Green fluorescent double-strand DNA (dsDNA); FL3: Red fluorescent single-strand (ssDNA); R1: Number of spermatozoa with DNA fragmentation; R2: Number of spermatozoa with dsDNA. Debris (bottom left corner) were excluded from the analysis and B. Density plot of sperm cells stained with FAM-DEVD-FMK/PI. Density plot shows viable, unstained spermatozoa in lower left quadrant (negative for FAM-DEVD-FMK and PI); live spermatozoa with caspase activity in lower right quadrant (FAM- DEVD-FMK positive only); and dead spermatozoa in upper right quadrant (positive for FAM-DEVD-FMK and PI) .

The DNA fragmentation index (DFI) was evaluated by the sperm chromatin structure assay (SCSA). The sperm suspension (1×10^6^ cells/mL) in PBS was mixed with an acid solution that consisted of Triton X-100 (0.1%), NaCl (0.15 mol/L), and HCl (0.08 N) at pH=1.4 for 40 seconds. Next, we added the staining solution that consisted of 6 mg/mL of acridine orange (AO) in a phosphate-citrate buffer. The sperm cells were subsequently analysed using a FACS Calibur flow cytometer. AO, when associated with single-strand DNA (ssDNA) and double-strand DNA (dsDNA) emits a red fluorescence detectable by a 670 band pass filter (Fl-3) and a green fluorescence was detected with 530/30 band pass filter (Fl-1), respectively. The DFI frequency dot plot is obtained from the ratio between the red and total (red plus green) fluorescence intensities (Fig .1A) (18).

#### Caspase 3 activity 

Caspase 3 activity was assessed using a FLICA Caspase Detection Kit (Catalog no. APT105; Chemicon.com; USA and Canada). Briefly, carboxyfluorescein (FAM-DEVDFMK) of the FLICA was used as the green fluorescence and PI as the red fluorescence (Fig .1B). 

For this evaluation, we diluted a sperm suspension 1×10^6^ cells/mL in phosphat buffer saline (PBS) and added 10 µl of prepared 30× FLICA reagent to the sperm suspension. The samples were incubated at 37°C with 5% CO_2_ for one hour in the dark. Then, the samples were washed with 2 ml of wash buffer (1X) and 2 µl PI was added to each tube. For each procedure, one tube was considered as a control. The results were analysed by a flow cytometer (17) . 

### Morphology 

The smears were prepared from washed samples on a glass slide and air-dried, then fixed in methyl alcohol and stained with Papanicolou staining. The percentage of sperm normality was recorded by calculation of 200 sperm cells under phase-contrast microscopy (CKX41, Olympus, Tokyo, Japan) at ×400 magnification (19).

### Flow cytometry analysis

Flow cemetery procedures were performed using a FACSCalibur (Becton Dickinson, San Jose, CA, USA) flow cytometer equipped with standard optics. A minimum of 10000 sperms were examined for each assay at a flow rate of 100 cells/seconds. The sperm population was gated using 90° and forward-angle light scatter to exclude debris and aggregates. The excitation wavelength was 488 nm supplied by an argon laser at 250 mW. Analysis of flow cytometry data was performed using FlowJo software (Treestar, Inc., San Carlos, CA, USA).

### Statistical analysis

All data were analysed by one-way ANOVA using SPSS V16.0 software. Statistical differences among various group means were determined by Tukey’s test. P<0.05 were considered to be statistically significant. Results are shown as mean ± SEM. 

## Results

### Sperm motility, velocity parameters, and normal morphology 

The mean percentage of motion characteristics, velocity parameters, and normal morphology of sperm in the different experimental groups (Table 1). The frozen control group had a significantly reduced percentage of TM and PM compared to the fresh group. There was significantly higher TM, PM, VAP, and VSL in the T60 group compared to the other freezing groups (P<0.05). The T90 group had a least significant percentage of TM, PM, VAP, VSL, VCL, and STR compared to the other groups. The percentage of normal morphology was significantly reduced in the control freezing group (6.81 ± 0.40) compared to the fresh group (10.70 ± 0.43). However, different induction times did not significantly impact the normal morphology of sperm in the cryopreserved groups.

### Phosphatidylserine translocation

The effects of sublethal stress induction times on the percentage of apoptotic and live sperm after cryopreservation (Table 2). We observed a significantly higher percentage of apoptotic (Annexin^+^/PI^-^ 19.45 ± 0.9) and dead (Annexin^+^/PI^+^, 21.41 ± 1.05) sperm in the control freezing group (1.84 ± 0.15) compared to the fresh group (3.91 ± 0.31). 

The percentage of live sperm (Annexin^-^/PI^-^ 55.63 ± 1.10) significantly increased in the T60 group compared to the other cryopreserved groups. T60 had a lower percentage of apoptotic rate (Annexin^+^/PI^-^ 12.29 ± 0.45) compared to the other cryopreserved groups (P<0.05). This parameter significantly increased in the T90 (33.40 ± 1.32) group compared to the other groups (P<0.05). 

### Caspase 3 activity and DNA fragmentation 

**Fig.2:**
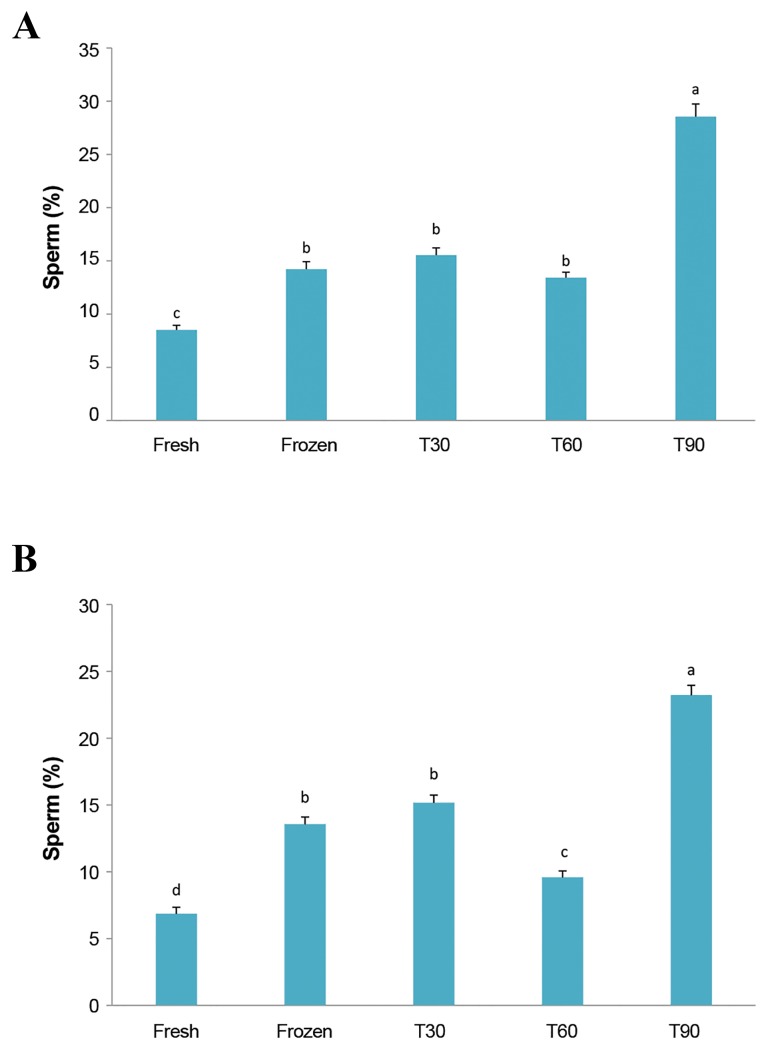
Effects of different induction times of sublethal stress on DNA fragmentation and caspase 3 activity. A. The percentage of DNA fragmentation between groups and B. The percentage of caspase 3 activity between groups. Data are mean ± SEM (n=20). a, b, c, d; Within the same columns are significantly different (P<0.05). Groups exposed to 0.01 μM nitric oxide (NO) during 30 minutes (T30), 60 minutes (T60), or 90 minutes (T90) before cryopreservation.

The percentage of DNA fragmentation and caspase 3 activity of sperm exposed to sublethal oxidative stress at different times (Fig .2A, B). The percentages of caspase 3 activity significantly increased in the control freezing group (13.52 ± 0.57) compared to fresh sperm (6.84 ± 0.50). The lowest percentage of caspase 3 activity was observed in the T60 group (9.54 ± 0.50) compared to the control freezing (13.52 ± 0.57), T30 (15.14 ± 0.59), and T90 (23.20 ± 0.75) groups. A higher significant percentage of DNA fragmentation was observed in the control freezing group (14.22 ± 0.70) compared to the fresh group (8.52 ± 0.43). T90 showed significantly increased DNA fragmentation (28.55 ± 1.19) compared to the other groups. For DNA fragmentation, there were no significant differences between different times of stress induction.

**Table 1:** Effects of different induction times of sublethal stress on sperm motility parameters and normal morphology


Groups	TM (%)	PM (%)	VAP (µm/s)	VSL (µm/s)	VCL (µm/s)	STR (%)	LIN (%)	Normal morphology (%)

Fresh	89.30 ± 0.93^a^	73.17 ± 1.35^a^	75.18 ± 1.20^a^	62.45 ± 4.3^a^	120.90 ± 4.5^a^	83.06 ± 0.85^a^	51.65 ± 1.70^a^	10.7 ± 0.43^a^
Frozen	51.60 ± 1.20^c^	32.55 ± 1.15^c^	48.75 ± 1.69^c^	35.29 ± 3.12^c^	90.91 ± 3.11^bc^	72.38 ± 1.45^b^	38.81 ± 1.65^b^	6.81 ± 0.40^b^
T30	49.28 ± 1.20^c^	30.31 ± 1.00^c^	44.20 ± 0.91^c^	31.70 ± 2.40^c^	82.63 ± 3.12^c^	71.71 ± 1.23^b^	38.36 ± 1.66^b^	6.73 ± 0.35^b^
T60	60.65 ± 1.15^b^	43.75 ± 0.95^b^	62.06 ± 1.63^b^	46.09 ± 3.32^b^	96.11 ± 3.11^b^	74.26 ± 1.00^b^	47.95 ± 1.60^a^	7.83 ± 0.29^b^
T90	33.31 ± 0.79^e^	18.25 ± 1.40^d^	32.95 ± 1.10^e^	22.11 ± 1.75^e^	61.95 ± 1.92^d^	67.10 ± 1.30^c^	35.69 ± 1.37^b^	6.54 ± 0.44^b^


Data are expressed as mean ± SEM (n=20). Groups were exposed to 0.01 μM nitric oxide (NO) for 30 minutes (T30), 60 minutes (T60), or 90 minutes (T90) before cryopreservation. ^a, b, c, d^; Within the same columns are significantly different (P<0.05), TM; Total motility, PM; Progressive motility, VCL; Curvilinear velocity, VSL; Straight line velocity, VAP; Average path velocity, LIN; Linearity, and STR; Amplitude of straightness.

**Table 2:** Effects of different induction times of sublethal stress on apoptotic levels in sperm


Groups	ANNEXIN^+^/PI^+^	ANNEXIN^+^/PI^-^	ANNEXIN^-^/PI^-^	ANNEXIN^-^/PI^+^

Fresh	3.91 ± 0.31^d^	1.84 ± 0.15^d^	83.31 ± 0.82^a^	10.91 ± 0.80^b^
Frozen	21.41 ± 1.05^b^	19.45 ± 0.9^b^	42.70 ± 0.95^c^	16.41 ± 0.9^a^
T30	22.70 ± 0.94^b^	21.28 ± 1.20^b^	40.60 ± 0.93^c^	15.39 ± 0.94^a^
T60	14.81 ± 0.84^c^	12.29 ± 0.45^c^	55.63 ± 1.10^b^	17.24 ± 0.82^a^
T90	34.29 ± 1.45^a^	33.40 ± 1.32^a^	19.94 ± 0.61^d^	12.34 ± 0.73^b^


Data are expressed as mean ± SEM (n=20). Groups exposed to 0.01 μM nitric oxide (NO) during 30 minutes (T30), 60 minutes (T60), and 90 minutes (T90) before cryopreservation. ^a, b, c, d^; Within the same columns are significantly different (P<0.05). PI; Propidium iodide, ANNEXIN^+^/PI^+^; Dead sperm, ANNEXIN^-^/PI^+^; Necrotic sperm, ANNEXIN^-^/PI^-^; Live sperm, and ANNEXIN^+^/PI^-^; Apoptotic sperm.

## Discussion

The main approach for sperm protection against cryoinjury is based on the use of antioxidants and cryoprotectants as defensive methods (20-22). Thus far, the antioxidant application is not completely sufficient to eliminate the damage caused by the freezing-melting process because sperm use only a small amount of added antioxidants during cryopreservation (23). The protective properties of antioxidants may be lost during processing and cooling (15). During the cryopreservation process, the concentration of free radicals is higher than antioxidants and often the amount of antioxidant added to the cryoprotectant is not enough (24). Limited achievements of the antioxidant strategies have encouraged researchers to develop a novel approach. This strategy is stress preconditioning of sperm before cryopreservation with mild, controllable stress. Recent studies have reported that application of various stressors such as hydrostatic pressure (7, 13), osmotic pressure (5, 12), and oxidative agents (15) at sublethal levels could improve the stress tolerance of sperm during cryopreservation. Previous studies have suggested that induction of mild stress may increase HSP expression and antioxidant protein levels in sperm (25). It seems that this response is principally dependent on the time and dose of this sublethal stress. 

In an animal study, Sharafi et al. (16) evaluated different induction times of sublethal stress with NO before cryopreservation. They observed that 75 and 120 minutes of stress significantly improved the quality of thawed semen compared to 30 and 90 minutes. An appropriate period for induction of sublethal stress could increase tolerance to cryo-stress. Several studies have reported improved sperm quality after exposure to NO (26-28). In the present study, we applied a low concentration of sublethal oxidative stress by using NO (0.01 µM) at 30, 60, or 90 minutes before cryopreservation. The quality parameters of thawed sperm were significantly higher when sublethal stress was applied 60 minutes before cryopreservation. Bock et al. (9) evaluated the effect of different induction times of sublethal stress with HHP (60 MPa) on mouse blastocysts. They reported that HHP treatment for 60 minutes caused significant upregulation of stress-related genes Azin1, Sod2, and Gadd45g, whereas the 120 minute treatment resulted in lower blastocyst formation and reduction in transcript quantity for all genes compared to the other times.

Sperm cells appear to need a suitable time for initiation of their defence system and dynamic mechanisms to adapt to environmental changes such as cryopreservation (13, 15). It has been reported that key factors that respond to sublethal stress include biosynthesis of stress-related proteins such as HSP and intracellular antioxidants that can maintain cellular homeostasis during environmental stress, resulting in protection of cells against cryoinjury (29, 30).

Of note, since sperm cells are regarded as transcriptionally inactive, the above proteins may be the result of posttranscriptional stabilization by sublethal stress (7, 25). In accordance with the results by Lefièvre et al. (31), our findings showed that human sperm under mild oxidative stress before cryopreservation required 60 minutes to adapt and regulate the response for protection against cryoinjuries. They reported that 60 minutes was a suitable time for induction of post-translational modifications like S-nitrosylation on human sperm proteins such as HSPs. In another study, it was suggested that the required time for modification of the protein profile of boar sperm under sublethal stress was 90 minutes before freezing (10). They observed that proteins like ubiquinol-cytochrome C reductase complex core protein 1, perilipin, and carbohydrate-binding protein AWN precursor increased in sperm cells following treatment with HHP for 90 minutes.

Mouse blastocysts treated with HHP (60 MPa) for 30 minutes showed significantly increased survival rates after freezing (32). Treatment of bovine blastocysts at 60 MPa for one hour before vitrification resulted in higher survival and hatching rates (33). Du et al. (8) reported that the best effect of HP treatment in porcine oocyte occurred one hour before vitrification. This discrepancy in the optimum time of stress induction was probably related to the differences in species, cells, and type of stressor. In the present study, induction of mild oxidative stress for 60 minutes reduced the percentage of apoptosis and caspase 3 activity. This time has appeared to be is appropriate for the biosynthesis or modification of stress related proteins (34). These proteins can block apoptotic signalling and improve sperm cryosurvival (34, 35). In our study, although NO had been able to prevent cryodamage, it could not improve DNA integrity after cryopreservation, further studies are necessary to evaluate this. 

This is the first assessment of the effects of different induction times of sublethal stress on frozen-thawed human sperm function. It requires further analyses and investigation to evaluate potential intermediate signalling components and their regulatory roles in human sperm biology and cryobiology.

## Conclusion

We observed that stress preconditioning of human sperm before cryopreservation with NO (0.01 µm) for 60 minutes could protect human sperm against cryo-injuries. Of note, the current study findings were obtained from normal and high quality sperm. More studies need to be undertaken to assess this hypothesis on other sperm (e.g., asthenozoospermia). Evaluation of the intermediate signalling pathway underlying this hypothesis should be of interest for future studies. This approach may improve sperm conservation protocols in assisted reproductive techniques.

## References

[1] Anger JT, Gilbert BR, Goldstein M (2003). Cryopreservation of sperm: indications, methods and results. J Urol.

[2] Di Santo M, Tarozzi N, Nadalini M, Borini A (2012). Human sperm cryopreservation: update on techniques, effect on dna integrity, and implications for ART. Adv Urol.

[3] Manee-in S, Parmornsupornvichit S, Kraiprayoon S, Tharasanit T, Chanapiwat P, Kaeoket K (2014). L-carnitine supplemented extender improves cryopreserved-thawed cat epididymal sperm motility. Asian- Australas J Anim Sci.

[4] de Paula TS, Bertolla RP, Spaine DM, Cunha MA, Schor N, Cedenho AP (2006). Effect of cryopreservation on sperm apoptotic deoxyribonucleic acid fragmentation in patients with oligozoospermia. Fertil Steril.

[5] Ozkavukcu S, Erdemli E, Isik A, Oztuna D, Karahuseyinoglu S (2008). Effects of cryopreservation on sperm parameters and ultrastructural morphology of human spermatozoa. J Assist Reprod Genet.

[6] Agarwal A, Durairajanayagam D, du Plessis SS (2014). Utility of antioxidants during assisted reproductive techniques: an evidence based review. Reprod Biol Endocrinol.

[7] Pribenszky C, Horvath A, Vegh L, Huang SY, Kuo YH, Szenci O (2011). Stress preconditioning of boar spermatozoa: a new approach to enhance semen quality. Reprod Domest Anim.

[8] Du Y, Pribenszky CS, Molnar M, Zhang X, Yang H, Kuwayama M (2008). High hydrostatic pressure: a new way to improve *in vitro* developmental competence of porcine matured oocytes after vitrification. Reproduction.

[9] Bock I, Losonczi E, Mamo S, Polgar Z, Harnos A, Dinnyes A (2010). Stress tolerance and transcriptional response in mouse embryos treated with high hydrostatic pressure to enhance cryotolerance. Cryo Letters.

[10] Huang SY, Pribenszky C, Kuo YH, Teng SH, Chen YH, Chung MT (2009). Hydrostatic pressure pre-treatment affects the protein profile of boar sperm before and after freezing-thawing. Anim Reprod Sci.

[11] Horvath A, Szenci O, Nagy K, Vegh L, Pribenszky C (2016). Stress preconditioning of semen before cryopreservation improves fertility and increases the number of offspring born: a prospective randomised study using a porcine model. Reprod Fertil Dev.

[12] Cole JA, Meyers SA (2011). Osmotic stress stimulates phosphorylation and cellular expression of heat shock proteins in rhesus macaque sperm. J Androl.

[13] Pribenszky C, Vajta G, Molnar M, Du Y, Lin L, Bolund L (2010). Stress for stress tolerance?. A fundamentally new approach in mammalian embryology. Biol Reprod.

[14] Dodaran HV, Zhandi M, Sharafi M, Nejati-Amiri E, Nejati-Javaremi A, Mohammadi-Sangcheshmeh A (2015). Effect of ethanol induced mild stress on post-thawed bull sperm quality. Cryobiology.

[15] Sharafi M, Zhandi M, Shahverdi A, Shakeri M (2015). Beneficial effects of nitric oxide induced mild oxidative stress on post-thawed bull semen quality. Int J Fertil Steril.

[16] Sharafi M, Zhandi M, Shahverdi A, Shakeri M, Nejati AE, Nejati JA (2015). The time effect of induced mild oxidative stress before freezing on the post-thawed sperm quality. JCT.

[17] Tavalaee M, Deemeh MR, Arbabian M, Nasr-Esfahani MH (2012). Density gradient centrifugation before or after magnetic-activated cell sorting: which technique is more useful for clinical sperm selection?. J Assist Reprod Genet.

[18] Hosseinifar H, Yazdanikhah S, Modarresi T, Totonchi M, Sadighi Gilani MA, Sabbaghian M (2015). Correlation between sperm DNA fragmentation index and CMA3 positive spermatozoa in globozoospermic patients. Andrology.

[19] Kruger TF, Ackerman SB, Simmons KF, Swanson RJ, Brugo SS, Acosta AA (1987). A quick, reliable staining technique for human sperm morphology. Arch Androl.

[20] Amidi F, Pazhohan A, Shabani Nashtaei M, Khodarahmian M, Nekoonam S (2016). The role of antioxidants in sperm freezing: a review. Cell Tissue Bank.

[21] Bateni Z, Azadi L, Tavalaee M, Kiani-Esfahani A, Fazilati M, Nasr- Esfahani MH (2014). Addition of Tempol in semen cryopreservation medium improves the post-thaw sperm function. Syst Biol Reprod Med.

[22] Azadi L, Tavalaee M, Deemeh MR, Arbabian M, Nasr-Esfahani MH (2017). Effects of Tempol and Quercetin on Human Sperm Function after Cryopreservation. Cryo Letters.

[23] Zhandi M, Sharafi M (2015). Negative effect of combined cysteine and glutathione in soy lecithin-based extender on post-thawed ram spermatozoa. Cell Tissue Bank.

[24] Agha-Rahimi A, Khalili MA, Nabi A, Ashourzadeh S (2014). Vitrification is not superior to rapid freezing of normozoospermic spermatozoa: effects on sperm parameters, DNA fragmentation and hyaluronan binding. Reprod Biomed Online.

[25] Pribenszky C, Vajta G (2011). Cells under pressure: how sublethal hydrostatic pressure stress treatment increases gametes’ and embryos’ performance. Reprod Fertil Dev.

[26] Hellstrom WJ, Bell M, Wang R, Sikka SC (1994). Effect of sodium nitroprusside on sperm motility, viability, and lipid peroxidation. Fertil Steril.

[27] Herrero MB, Gagnon C (2001). Nitric oxide: a novel mediator of sperm function. J Androl.

[28] Yang MG, Yang Y, Huang P, Zheng SL, Fan AL, Cheng XD (2005). Sodium nitroprusside facilitates human sperm capacitation and acrosome reaction. Zhonghua Nan Ke Xue.

[29] Senf SM, Dodd SL, McClung JM, Judge AR (2008). Hsp70 overexpression inhibits NF-kappaB and Foxo3a transcriptional activities and prevents skeletal muscle atrophy. FASEB J.

[30] Huang SY, Kuo YH, Lee YP, Tsou HL, Lin EC, Ju CC (2000). Association of heat shock protein 70 with semen quality in boars. Anim Reprod Sci.

[31] Lefièvre L, Chen Y, Conner SJ, Scott JL, Publicover SJ, Ford WC (2007). Human spermatozoa contain multiple targets for protein Snitrosylation: an alternative mechanism of the modulation of sperm Sublethal Stress Improves Cryotolerance in Sperm function by nitric oxide?. Proteomics.

[32] Pribenszky C, Molnar M, Cseh S, Solti L (2005). Improving post-thaw survival of cryopreserved mouse blastocysts by hydrostatic pressure challenge. Anim Reprod Sci.

[33] Siqueira Filho E, Caixeta ES, Pribenszky C, Molnar M, Horvath A, Harnos A (2011). Vitrification of bovine blastocysts pretreated with sublethal hydrostatic pressure stress: evaluation of post-thaw *in vitro* development and gene expression. Reprod Fertil Dev.

[34] Kultz D (2005). DNA damage signals facilitate osmotic stress adaptation. Am J Physiol Renal Physiol.

[35] Garrido C, Brunet M, Didelot C, Zermati Y, Schmitt E, Kroemer G (2006). Heat shock proteins 27 and 70: anti-apoptotic proteins with tumorigenic properties. Cell Cycle.

